# UNCOVERING THE POSITIVE ASPECTS OF CAREGIVING: A PROFILE OF CAREGIVERS’ DEMOGRAPHIC AND CARE CONTEXTS

**DOI:** 10.1093/geroni/igac059.2701

**Published:** 2022-12-20

**Authors:** Yeonjung Lee, Alex Bierman

**Affiliations:** Chung-Ang University, Seoul, Seoul-t’ukpyolsi, Republic of Korea; University of Calgary, Calgary, Alberta, Canada

## Abstract

Burden and benefits of caregiving experiences can coexist. The objective of this research is to describe and compare the predictors of the two intertwined caregiving experiences. This study examines how the variations in caregiving experiences can be explained in terms of both positive and negative aspects of caregiving, respectively by demographic characteristics and care related contexts. The Caregiving, Aging, and Financial Experiences study is a national survey intended to examine social conditions and well-being among a representative sample of 4,010 Canadians between age 65 and 85. Within the sample, 1,641 informal caregivers are the focus of the current analysis. Scales of positive and negative caregiving experiences are employed. Findings based on principal axis factor analysis shows that there is clear separate factor loadings between the positive and negative caregiving experiences. Subsequent seemingly unrelated regression analysis shows that there are similarities as well as differences in predictors between the two caregiving experiences. Lastly, the variance explained differs markedly between the two measures, with over 26% of the variance in negative caregiving accounted for by demographic and caregiving factors, but less than 4% of the variance in positive caregiving. This study demonstrates that positive aspects of caregiving is not simply the flip-side of negative caregiving. Standard predictors do not sufficiently explain positive caregiving as well as negative caregiving. Consequently, greater attention to factors that account for positive aspects of caregiving is warranted for an inclusive understanding of caregiving experiences.

